# Facilitating support groups for siblings of children with neurodevelopmental disorders using audio-conferencing: a longitudinal feasibility study

**DOI:** 10.1186/s13034-015-0041-z

**Published:** 2015-04-07

**Authors:** Sheryl Gettings, Fabia Franco, Paramala J Santosh

**Affiliations:** Florence Nightingale Faculty of Nursing and Midwifery, King’s College London, London, UK; Department of Psychology, School of Science and Technology, Middlesex University, London, UK; Centre for Interventional Paediatric Psychopharmacology (CIPP), Maudsley Hospital; and Department of Child and Adolescent Psychiatry, Institute of Psychiatry, Psychology and Neuroscience, King’s College London, London, UK

**Keywords:** Sibling, Support group, Behavioural problems, Telemedicine, Young carer, Autism Spectrum Disorder, Chronic illness, Neurodisability, Complex neurodevelopmental disorders

## Abstract

**Background:**

Siblings of children with chronic illness and disabilities are at increased risk of negative psychological effects. Support groups enable them to access psycho-education and social support. Barriers to this can include the distance they have to travel to meet face-to-face. Audio-conferencing, whereby three or more people can connect by telephone in different locations, is an efficient means of groups meeting and warrants exploration in this healthcare context. This study explored the feasibility of audio-conferencing as a method of facilitating sibling support groups

**Methods:**

A longitudinal design was adopted. Participants were six siblings (aged eight to thirteen years) and parents of children with complex neurodevelopmental disorders attending the Centre for Interventional Paediatric Psychopharmacology (CIPP). Four of the eight one-hour weekly sessions were held face-to-face and the other four using audio-conferencing. Pre- and post-intervention questionnaires and interviews were completed and three to six month follow-up interviews were carried out. The sessions were audio-recorded, transcribed and thematic analysis was undertaken.

**Results:**

Audio-conferencing as a form of telemedicine was acceptable to all six participants and was effective in facilitating sibling support groups. Audio-conferencing can overcome geographical barriers to children being able to receive group therapeutic healthcare interventions such as social support and psycho-education. Psychopathology ratings increased post-intervention in some participants. Siblings reported that communication between siblings and their family members increased and siblings’ social network widened.

**Conclusions:**

Audio-conferencing is an acceptable, feasible and effective method of facilitating sibling support groups. Siblings’ clear accounts of neuropsychiatric symptoms render them reliable informants. Systematic assessment of siblings’ needs and strengthened links between Child and Adolescent Mental Health Services, school counsellors and young carers groups are warranted.

## Background

A growing number of children are being diagnosed with chronic illnesses and disabilities, with epidemiological research showing that 12% to 14% of the child population experience mental health problems [1]. The prevalence of Autism Spectrum Disorder (ASD) and Attention Deficit Hyperactivity Disorder (ADHD) in the United Kingdom (UK) is currently estimated to be 1.7% and 1.4% respectively [2]. It has long been recognised that well siblings of children with chronic illness are potentially the most overlooked and unhappy family member [3]. Siblings of children with chronic illness or ASD are at increased risk for negative psychological adjustment and negative psychological effects; in particular for internalising behaviours such as anxiety and depression [4,5]. Negative manifestations include anger, resentment, frustration, loneliness, sadness, worry, fear, over-identification, feeling envious or jealous, confusion, secluding themselves and regression [6,7]. These could result from less parental attention, excessive demands placed upon siblings and a perception of unequal treatment [8,9]. The nature and degree of need their affected brother/sister has should be taken into account when considering the impact on siblings. The greater the care-giving demands and hence parental attention the child with a chronic illness needs, the more the siblings are negatively affected [5].

Means of early intervention are necessary to improve the psychological wellbeing and mental health of children and young people at risk of negative psychological effects [10]. The needs of siblings should, for this reason, be provided for as part of a package of services for the child with a disability [11]. In the UK, the Department for Children, Schools and Families (DCSF) and the Department of Health (DH) set out guidance for commissioning services and early intervention to improve the psychological wellbeing and mental health of children and young people [10]. Parenting support interventions are recommended in the guidance, however there is no specific reference to interventions for siblings. Potential positive outcomes for siblings such as increased empathy, personal maturation, enhanced self-concept, increased independence and a unique world-view, should be acknowledged when planning care [6,12-14]. Through effective interventions, the care and outcomes of well siblings can be enhanced [14].

Siblings are involved in caring for their brother or sister with a disability and have a sense of increased responsibility and pressure to take care of and worry about their family [6,15,16]. As well as being affected directly and indirectly by stress on the family, in particular on their parents, siblings of children with autism are vulnerable to emotional stress through being seen as and seeing themselves as responsible for unusually high levels of assistance within the home [16]. The importance of identifying children with inappropriate caring responsibility is highlighted in the ‘No Health Without Mental Health’ strategy [17]. However, given the nature of care being relational or involving behaviour management, recognising inappropriate caring responsibilities in the context of being a sibling of a child or young person with behavioural difficulties is complex. Some siblings are ‘young carers’, defined as ‘children and young people under 18 who provide or intend to provide care, assistance or support for a family member’ [18]. Young carers carry out substantial caring tasks regularly and assume a level of responsibility that would usually be associated with an adult. The person receiving care is usually a parent, sibling, grandparent or relative with a disability, chronic mental or physical health problem, connected with a need for care, support or supervision [18].

Based on parental reports, the 2011 Census reported there were 177,918 young carers in England and Wales [19], an underestimate, as it is not based on self-report [20]. A more realistic estimate is 700,000 young carers in the UK [21]. The number of those caring for a sibling is as yet unspecified. In addition, the nature of caregiving might not be fully appreciated. There is a need to recognise young carer status in siblings involved in either the physical care or in managing behaviour sometimes associated with neurodevelopmental disorders in order for them to receive the support they require. It is particularly significant when dealing with challenging behaviours, for example, aggression towards family members, or with self-injury behaviours. A comprehensive understanding of the impact of challenging behaviours on siblings would assist in identifying the support siblings require [22]. Young carers are among those who contact the National Society for the Prevention of Cruelty to Children 24 hour helpline ChildLine [23]. Notably, during 2011–12, matters relating to family relationships accounted for the highest percentage of phone calls, emails and online chats to ChildLine. These findings have led to an additional category being measured addressing issues around being a young carer [24]. More work must be done to recognise young carers, identify their needs and to provide the support they deserve.

The ability for healthy siblings to successfully adjust may be moderated by their access to social support and by the severity of their brother or sister’s autism [25]. Support for siblings can be provided through sibling support groups which offer emotional and significant social support, provide psycho-education and enable children to widen their social network thereby potentially strengthening their resilience [26-29]. Potential outcomes include reduced anxiety [30] and positive affects to siblings’ well-being and self-esteem [31]. In addition, sibling support groups can facilitate the identification of any need for additional services [31]. Even in adulthood, the importance of support groups for siblings of individuals with psychotic illness has been highlighted with there being a need for continued research [32]. However there is a lack of systematic evaluation of sibling support group interventions and clinically meaningful measures must be applied [33].

To ensure siblings have access to support, healthcare providers have a duty to address barriers to them accessing services. In the UK and internationally, the distance families have to travel to a national specialist service, can be a barrier to them accessing support needed [33]. Reasons for this can include parents’ difficulty with organising care for the sibling’s brother or sister while they accompany the sibling to the venue and the financial impact of travel costs. Previous studies reporting interventions to support siblings have not specifically addressed barriers to them accessing support. It is important to identify acceptable and feasible means of overcoming the barrier of distance to ensure vulnerable individuals and groups can access psychosocial support. This issue has global relevance with there being 18 million children displaced by conflict, economic pressures or natural disaster. There is a need for effective interventions for those who experience psychological distress, feelings of isolation and assume young carer roles [34]. The United Nations Children’s Fund [35] reports that distress levels of children living in urban poverty are greater than the national average, showing that not all urban residents can easily access services. It is crucial to design and deliver services in ways that enable them to be accessed by all residents who require them in every region of every nation.

Telemedicine has significant potential in overcoming the barrier of geographical distance. Audio-conferencing can be used as a telemedicine technique in place of face-to-face (F2F) meetings with three or more people connecting by telephone in different locations. Weiner et al. [36] reported the usefulness of telephone support groups with adults and one with girls who were HIV positive. To the best of our knowledge, the effectiveness of the use of audio-conferencing with siblings, children or young people has not been explored. Adult participants have reported benefits from accessing social support through telephone support groups including reduced isolation, increased knowledge and confidence and anonymity if wanted [36-41].

The acceptability of this means of accessing social support for children and young people requires exploration. Mobile phone technology is increasingly being used in developed and developing countries [42], and it is be-coming common for young people to use texting or web-based social networking sites. The acceptance of technology allowing for seeing one another’s face ‘on-screen’ in ‘real time’ has not yet reached the same proportions in health care delivery. The need to keep mobile devices at arm’s length in order to get a good facial picture can lead to the loss of privacy, and they may have to speak louder because of the distance they are from the phone. In addition, seeing each other could in some cases be ‘too close’ for them and the alternative of connecting by voice within an anonymous context could allow for more openness.

The aims of the study were i) to explore the acceptability and feasibility of audio-conferencing as a method of facilitating support groups for siblings of children with neurodevelopmental disorders, ii) to explore whether the participants can discuss issues that concern them via audio-conferencing, to demonstrate that this modality can be used for therapeutic work and iii) to explore the impact of facilitative support groups after three to six months. The above aims would be demonstrated by showing that a) all siblings engage in group sessions whether via audio-conferencing or F2F, b) siblings are able to share their experiences and uppermost concerns with each other, their ideas for problem-solving and to access psycho-education, c) siblings and parents give positive evaluations of the sibling support group, and d) siblings keep in touch with one another three to six months after the support group, demonstrating an increased support network. In this study, it was important to remain aware of several outcomes about conducting a sibling support group and the potential effect it may have on siblings’ quality of life. There may be no change, improvement, or there may be an increased awareness of the challenges in their lives. This in-depth longitudinal feasibility study aims to provide a robust model for future research.

## Methods

### Ethics

Ethics approval was obtained from the hospital Research Ethics Committee and Middlesex University’s psychology ethics panel. Parents who agreed to receive further information about the research were given two weeks to consider taking part and advised that either they or their child could contact the researcher and ask questions about the study at any time. Informed consent and assent were obtained from parents and siblings after giving them full opportunity to consider taking part and having the opportunity to ask questions. For those siblings agreeing to take part, a letter was sent to their General Practitioner (family doctor), with permission from their parents, informing them that the sibling was going to take part in the sibling support group. If at any point during or following the study, the sibling required more support than could be provided in the sibling support group, researchers would refer the sibling to their General Practitioner for assessment. The sharing of sensitive information would be managed appropriately as facilitators were experienced clinicians.

### Design

The support group participants were siblings of patients being treated at the Centre for Interventional Paediatric Psychopharmacology (CIPP), a national specialist Child and Adolescent Mental Health Service (CAMHS) in the UK. All patients had complex neurodevelopmental disorders involving at least two co-morbid conditions such as ASD, ADHD, obsessive compulsive disorder, oppositional defiant disorder or anxiety disorders. The sibling support group consisted of weekly one-hour sessions for eight consecutive weeks and were followed up three to six months after the last session.

In order that siblings and parents could evaluate audio-conferencing as a means of the sibling support group being facilitated, it was important that an equal number of each type of session (four held F2F and four using audio-conferencing) was experienced. Sessions one, two, five and eight were held F2F, and the other four took place using audio-conferencing. Siblings were then able to meet in person on two occasions before attempting to talk in a group over the telephone. Holding the final session F2F would allow siblings to complete the support group process in person and say their farewells F2F. Session five was identified as a F2F session as it was mid-point between the four audio-conferencing sessions.

Structured and semi-structured pre- and post-intervention paper-form questionnaires were administered and pre- and post-intervention semi-structured and unstructured interviews were carried out with siblings and parents, as indicated in Table 1. Data triangulation was used to promote quality in the research and enrich understanding [43,44]. Triangulation involved combining different sources of data that converged on a single construct e.g. data obtained from semi-structured interviews was combined with data collected from group discussions and with semi-structured questionnaires. Additionally, quantitative data could be considered within the context of qualitative data. Minimal resources were available for this pilot study and the inclusion of a control group was not considered essential for its purposes.

**Table 1:** **Pre-and post-intervention data collection tools**

**Data collection tool**	**Format**	**Siblings**		**Parents**	
		**Pre-intervention**	**Post-intervention**	**Pre-intervention**	**Post-intervention**
Profile of Neuropsychiatric Symptoms	Paper-form			✓	
Sibling's Views Questionnaire	Semi-structured face-to-face interview	✓	✓		
Strengths and Difficulties Questionnaire	Paper-form	✓	✓	✓	✓
Paediatric Quality of Life Inventory™ Version 4.0	Paper-form	✓	✓	✓	✓
Evaluation Questionnaire	Paper-form		✓		✓
Follow-up interview	Unstructured face-to-face interview		✓		✓

### Participants

Participants were a convenience sample of six siblings (five girls and one boy) aged eight to thirteen years (all were younger than their affected sibling). All siblings were accompanied by parents. Five mothers and one father took part. Siblings recruited into the study had affected brothers or sisters being treated in the CIPP. An autism spectrum disorder was present in all six of the siblings’ affected brothers/sisters. Apart from this, four of the affected brothers/sisters also had ADHD, four had a mood disorder, and two had obsessive compulsive disorder. One each had Down’s syndrome, oppositional defiant disorder, enuresis, visual impairment, harmful use of cannabis, multiple anxiety disorders or phobias. Only one sibling who took part was a member of a young carer organisation. All participants were English-speaking. Their affected brothers/sisters were aged 11 to 13 years and had received their diagnoses between two and four years prior to the study commencing.

### Procedure

Seven out of fifteen parents and siblings invited agreed to participate. Eight parents who chose not to participate gave the following reasons i) difficulty travelling to the hospital for the F2F sessions (n = 3), ii) the cost of travelling to the hospital for the F2F sessions (n = 1), iii) difficulty finding childcare for the affected brother or sister during the F2F sessions (n = 2), iv) the parent having to work in the evenings (therefore being unable to bring the sibling to attend the F2F sessions) (n = 1), v) the timing of the session clashed with other regular after-school activity already arranged (n = 1), or vi) considered not needed (n = 2). One sibling, 11 years of age, chose not to participate due to concerns about being recorded, completing questionnaires and missing some school to travel a long way to reach the hospital for the F2F sessions. Another sibling (14 years of age) withdrew after the first session as he considered that he had found his own way of coping. He stated he would have liked the opportunity of attending a support group when he was younger.

Participants lived 10 to 351 kilometres from the hospital clinic. Four of them lived within 100 kilometres of the clinic, and two lived further away (214 km and 351 km) but were keen to participate. Siblings expressed disappointment if they had to miss any group meetings, which indicated their keenness to take part.

### Data collection

#### Pre- and post- intervention questionnaires

Pre-intervention questionnaires were administered at the start of the first support group session and post-intervention questionnaires at the end of the final support group session. A semi-structured Sibling’s Views Questionnaire (SVQ) was designed specifically for this study consisting of six questions and administered by interview with each sibling individually (face-to-face) and audio-recorded. The interviews took 20 to 30 minutes each and were conducted by SG, a Clinical Nurse Specialist, experienced in clinical interviews and neuropsychiatric assessment. PS provided supervision, discussed transcripts and helped interpret the information obtained. The SVQ elicited information about siblings’ uppermost concerns with regard to their brother/sister’s behaviour, the impact of this behaviour on them and their family, whom they talk to about it and what they would like from being in the sibling support group. The post-intervention SVQ revisited the information each of the siblings raised in their SVQ pre-intervention, to establish any changes that had taken place.

Sibling and parent versions of a semi-structured paper-format Evaluation Questionnaire, consisting of 11 questions, were also designed for use in this research. These took 15 to 20 minutes to complete and ascertained the difficulty or ease with which siblings managed to use the telephone to join in the support group sessions, what they liked and disliked about the F2F and audio-conferencing sessions, what (if anything) they think was of benefit to them about being in the sibling support group and what they would have changed. The content of the SVQ and EQ was developed through expert consensus of professionals working in the CIPP, where audio-conferencing has been used routinely as part of clinical assessment for over 10 years. Input was also obtained from patients and parents who use audio-conferencing in the clinic. The instruments were not formally pilot-tested but were primarily used to obtain qualitative information.

Pre-intervention, siblings’ parents completed a paper-format Profile of Neuropsychiatric Symptoms questionnaire (PONS) providing a baseline measure of the frequency and severity of outpatients’ symptoms at the start of the study [45]. The PONS scale covers child- or parent-rated symptoms of neurodevelopmental disorders such as ADHD and ASD, alongside symptoms of psychoses, bipolar disorder, anxiety and depression and takes 10–12 minutes to complete. The PONS is completed routinely by parents for the affected brother/sister’s follow-up appointments. It has 31 items with frequency and impairment ratings, using a 6-point Likert scale, and has good criterion validity [45]. The PONS scales are currently incorporated into the HealthTracker™, a health-monitoring platform and are used in different clinical and research settings across Europe [46]. The PONS was used to enable a direct comparison between the symptom profile reported by parents and the behaviours the siblings stated they were most concerned about in the SVQ. Through the process of seeking ethical approval, it was decided that siblings would not be asked to complete a PONS in relation to their brother or sister. The SVQ for this reason served as a suitable alternative data collection tool through which to obtain siblings’ report of their brother/sister’s behavioural difficulties. The SVQ ascertained siblings’ uppermost concerns. Acceptability and feasibility of audio-conferencing would be demonstrated through siblings feeling able to go on to share their concerns in the sibling support group sessions.

The paper version of the Strengths and Difficulties Questionnaire (SDQ) [47] was used as a measure of social, emotional and behavioural functioning pre- and post-intervention to enable changes to be identified. There are 25 items in the SDQ, divided into five scales measuring emotional symptoms, conduct problems, hyperactivity-inattention, peer problems and pro-social behaviour [48]. The impact supplement was included for both the siblings and the parents whereby participants are asked if they think the child has any difficulties in the areas of emotions, concentration, behaviour or getting on with other people. Through completing these, aspects of social, emotional and behavioural functioning could be compared with data obtained through the SVQs and themes emerging through discussions during the sibling support group sessions. The SDQ is widely used for children aged four to sixteen years. Its validity and reliability is very well established [48,49]. The PONS measure is used in the clinical context preceding follow-up appointments. For this reason siblings’ parents were familiar with it.

To establish changes to siblings’ quality of life following being part of the sibling support group, the Pediatric Quality of Life Inventory™ Version 4.0 (PedsQL™ 4.0) was used pre- and post-intervention (paper-format). The PedsQL™ 4.0 is a standard form 23-item quality of life questionnaire consisting of four Generic Core Scales: Physical Functioning, Emotional Functioning, Social Functioning and School Functioning [50]. Child report and parent versions were administered pre- and post-intervention. Varni et al*.* [51] documented that the PedsQL™ 4.0 is a reliable and valid measure of health-related quality of life. Its suitability for use in clinical trials, research, clinical practice school health settings and community populations has been demonstrated [51]. Data collected using the PedsQL™ 4.0 could be compared with that obtained from the SDQs and themes emerging from the support group sessions.

#### Support group sessions

Session themes were identified and structured workshop activities and materials were adapted for use from existing models [6,52]. Mechanisms through which therapeutic factors already identified in support groups could be cultivated were incorporated across the eight sessions i.e. group cohesiveness and installation of hope [53,54]. It was important to include ‘ice-breaker’ exercises and encourage group cohesion from the outset and exercises facilitating this were incorporated into the first two sessions. Given siblings’ need for psycho-education, session three was dedicated to providing siblings with the opportunity to gain a better understanding of their brother/sister’s diagnoses involving the opportunity for them to ask questions. Session four focused on matters relating to school to enable siblings to share their experiences at school including talking about friendships. The session provided them with the opportunity to discuss the impact on them from having a brother or sister with complex neurodevelopmental disorders. The fifth session was based on the theme ‘Leisure time’ that allowed siblings to share stories about recreational activities and daily family life. Session six focused on problem-solving, with a view to enabling siblings to enhance coping–mechanisms they might already have developed and add to those by sharing ideas with one another. This allowed the potential for siblings to recognise their strengths and build resilience. The theme of the penultimate session focused on their thoughts about the future including their hopes for their brother/sister and their dreams. An outline of the purpose and content of the weekly sessions is included in Table 2.

**Table 2:** **Outline of support group session focus and purpose**

**Session**	**F2F or AC**	**Focus**	**Group cohesion**	**Psycho-education**	**Problem solving**	**Instillation of hope [** 54 **]**	**Assessment**
**1**	**F2F**	**Explanations, questionnaires and introductions**	✓				Q* SI**
		The consent process was completed. Participants were introduced to one another, ‘Ground Rules’ were agreed and confidentiality was discussed. The procedure for joining the conference call was explained and an ‘instruction’ sheet for this was provided. Any questions parents and siblings had were addressed. Pre-intervention questionnaires were completed. Name badge-making and informal games were played, supervised by research assistants, as ‘ice-breaker’ activities. Permission was obtained for a sibling group photo for use by siblings to help them remember ‘who was who’ in particular during the sessions using audio-conferencing to ‘put a face and name to the voice’.					
**2**	**F2F**	**Getting to know each-other**	✓				
		A printed copy of the Ground Rules and the sibling group photo were circulated. Siblings wore the name badges they had made in the first session.					
		The ‘Human Bingo’ game and the ‘Strengths and Weaknesses’ activities [6] were carried out as ‘ice-breaker’ activities. This stimulated further discussion, mediated by the researchers. Time was taken out from discussion for refreshments and this allowed siblings to chat together in a less structured way.					
**3**	**AC**	**Understanding your brother’s or sister’s illness**		✓			
		Psycho-education was provided by the researchers whereby siblings could ask questions and discuss their brother/sister’s conditions. To facilitate fairness in terms of siblings’ equal participation and opportunity to talk, the researchers used a wipe-board to keep track of which siblings were speaking up. It was agreed that a printed information sheet would be compiled summarising the information on the main common diagnoses discussed and this was posted to each of the siblings.					
**4**	**AC**	**School matters**		✓	✓		
		Any further questions the siblings had about their brother/sister’s diagnoses were addressed. Researchers facilitated discussion around the theme of matters relating to school e.g. getting to school, doing school work including homework and getting on with peers at school. Researchers facilitated a problem-solving outlook to address issues that arose by encouraging siblings to share ideas for addressing challenges they spoke about.					
**5**	**F2F**	**Sharing stories about recreation time**	✓	✓	✓		
		The ‘Time Capsule’ activity [6] was used to facilitate discussion about leisure time. As parents requested feedback on issues that had arisen, it was agreed with the siblings that some key areas would be discussed in general terms with parents after the session. During that time, siblings engaged in ‘free chat’ with each other, having further refreshments, drawing and playing together.					
**6**	**AC**	**Sharing concerns and solutions**	✓		✓	✓	
		The ‘Aunt Blabby’ exercise [6] was used to demonstrate and encourage the siblings’ problem-solving skills and facilitate the sharing of concerns. Having posted out fictitious ‘agony aunt’ letters to siblings prior to the session, siblings took turns to read out their letter(s) during the conference call session and discussed as a group their own experiences and ideas for solutions.					
**7**	**AC**	**Talking about opportunities and thinking about the future**				✓	
		The Dream Cloud exercise was used as a means of siblings considering their aspirations and hopes for the future [52].					
**8**	**F2F**	**De-briefing and farewells and evaluation**				✓	Q*, SI**
		Group de-briefing took place with the siblings and the ‘Compli-note’ exercise was used as a facilitative tool for them to share positive thoughts about each other [52].					
		Post-intervention questionnaires were administered to siblings and to parents. The post-intervention SVQ semi-structured interviews were also carried out. Finally, further de-briefing with all siblings and parents took place.					

*Q = questionnaires, **SI = semi-structured interviews, F2F = face-to-face, AC = audio-conferencing.

The sessions for which siblings met F2F were held in the hospital school room. Arrangements were in place for privacy and confidentiality to be maintained during the audio-conferencing sessions. All sessions (F2F and audio-conferencing) took place on a weekday evening. A Consultant Child Psychiatrist (PS) and a Clinical Nurse Specialist (SG), both of whom are experienced clinicians, facilitated all of the group sessions. All sessions were audio-recorded, however technical difficulties resulted in no audio-recording for sessions two and three. Upon discovering this, detailed notes were written within 24 hours of the sessions having taken place, by both researchers, based on notes taken during the sessions. In all other instances, audio-recordings were transcribed by the Clinical Nurse Specialist.

#### Follow-up interviews

Each sibling was invited to an unstructured individual interview together with their parent(s), to take place three to six months following the final support group session. Examples of core questions asked in the follow-up interviews are ‘Do you still keep in touch with anyone from the sibling support group?’; ‘What did you get out of the sibling support group?’ and ‘What has changed since you attended the sibling support group?’. The interviews were individually tailored for each sibling and their parents and included a de-briefing process, the provision of feedback from researchers and opportunity for any questions to be asked by siblings and parents. Unstructured interviews were considered most appropriate to allow the flexibility for the siblings and parents to be as frank and open as they would like.

#### Data analysis

Thematic analysis was used to analyse data from the pre- and post-intervention SVQs, Evaluation Questionnaires, support group sessions and follow-up interviews. This evaluation was carried out by the Clinical Nurse Specialist (SG) and involved line-by-line coding, focused coding and synthesis of data [55,56]. Changes in pre- and post-intervention SDQ and PedsQL™ 4.0 data were compared. Statistical analyses were not carried out due to the small sample size.

## Results

### Attendance

There were only three occasions of non-attendance out of a possible 48 episodes. Two of the siblings could not attend one of the F2F sessions due to there being an exacerbation of their brother/sister’s behavioural difficulties whereby their parents could not enable the sibling to attend. One sibling could not attend one F2F session due to a pre-planned school trip taking place at that time. This high attendance (93%) indicated excellent engagement with support group sessions which is a marker of acceptability. Notably, the sessions siblings were unable to attend were F2F sessions.

### Sibling's Views Questionnaire

#### Siblings’ concerns, pre-intervention

Autism spectrum disorder symptoms featured in the concerns of all six siblings. Four siblings had concerns about their brother/sister’s aggression (physical, verbal or both) and three of the siblings had concerns featuring their brother or sister’s circumscribed interests. Two siblings had concerns involving their brother/sister’s explosive rage, anti-social behaviour, oppositionality or poor empathy. They reported that anti-social behaviour or aggression from their brother or sister was directed towards them, their parents, other siblings in the family or towards other children. Some of the aggressive behaviour which siblings described had resulted in physical injury to them and more serious physical injuries to their parents. One sibling described their affected brother’s impulsive outbursts of aggression being unprovoked and unpredictable and stated these incidents had been happening about once every two months for the last two years. In the case of one sibling, physical aggression was reported as happening for 10 years, at a current frequency of three times per week. Another sibling recalled aggression from her sister taking place over the previous three years at a current frequency of twice per day. One sibling described her brother’s behaviour when he was under the influence of illegal substances and three of the siblings had concerns which related to their sibling’s ADHD symptoms. Three of the siblings indicated awareness of ‘triggers’ or patterns to their brother/sister’s behaviour.

Siblings voiced their emotional responses to their affected brother/sister’s behaviour included fear, anger, upset, feeling hurt, a sense of injustice, worry or shock. One sibling expressed a fear of challenging their brother/sister due to predicting they would get upset or that it would trigger their challenging behaviour. One sibling reported being pre-occupied by their concerns about their sister during in the day and another sibling stated it was difficult to concentrate and that she got headaches. Siblings chose to raise concerns that had occurred over different ranges of time and on differing numbers of occasions. They elected to mention behaviours that had been happening repeatedly over a number of years and events that were ‘one-off’ behaviours that had emerged recently, about which they were concerned and confused.

Comparing parents’ PONS responses with siblings’ pre-intervention SVQ responses established that siblings are competent in identifying and articulating the behavioural difficulties in their affected brother/sister indicating that siblings can be reliable informants.

#### Siblings’ concerns, post-intervention

Post-intervention, all six siblings reported a reduction in the severity of at least one of their concerns. Reasons they gave for this were i) having increased understanding about their brother/sister’s diagnoses, ii) there being improvements in the management of their brother/sister’s behaviour, iii) a parent taking a firmer approach if their brother/sister hurt them, or iv) the sibling adopting new ways of coping with their brother/sister’s behaviour or through telling their parents about the problem (i.e. having not done so pre-intervention). Three siblings reported at least one of their main concerns had increased because of worsening symptoms e.g. low mood in their brother/sister sometimes resulted in worsening of their brother/sister’s behaviour directly towards them.

#### Talking to people about their concerns pre-intervention

Four siblings spoke to their parents about their concerns although for one of them that didn’t include talking to them about their brother’s self-harming behaviour, which instead they spoke to an imaginary friend about. One of the two siblings who didn’t already talk to her parents about her concerns did not state that she wanted to talk to them at that stage. The other sibling who did not talk to her mother about her concerns, talked to her older (well) sister and to a friend her own age. Referring to her affected sister, she stated “Mum notices her hit me at the time but we don’t talk about it. She’s [mother] too busy to talk. I ask her and she says ‘I’m busy’”. Three siblings referred to experiencing difficulties or having to be careful explaining their brother/sister’s difficulties to their peers. One sibling said she would like to talk to friends “but friends go a bit weird about it…. They don’t keep it a secret, and it becomes a big thing”. Another sibling stated “I tell my friend. I only tell my best friend because she understands me”.

#### Talking to people about their concerns post-intervention

Four of the siblings reported they talk on the telephone to at least one other sibling from the sibling support group outside of the support group sessions. Siblings who didn’t talk to their parents about their concerns pre-intervention had started to do so. In addition, three siblings spoke to more family members or adults (e.g. aunts, uncles, step-parent, school teacher, and parents’ friends). One sibling noted she would now also like to be able to talk about her concerns to children in her class at school.

#### Outcomes siblings said they wanted from the support group

Three siblings said they wanted information about their brother/sister’s illness including the prognosis. One sibling stated “I’d like to be told what’s going on and have explanations. I get told stuff but not explanations”. Two siblings wanted to know how other young people in this position think or “deal with life having a disabled brother or sister”. One sibling stated she wanted to make friends through the support group.

Post-intervention, all six siblings said they had gained what they wanted from the support group. Those who wanted information about their brother/sister’s illness said this had been achieved, but they would like to know more, including on an on-going basis from their parents. One sibling who wanted to know what it was like for others in the same position as them stated “I know I’m not exactly alone and there are other people that have the same difficulties as me”. One stated she appreciated the support group giving her “time away” from the situation at home. One sibling felt more empowered to voice her concerns to her mother post-intervention.

### SDQ

Pre- and post-intervention self- and parent-report sibling SDQ Impact Scores indicated either the same level of impact or an increased level of impact was reported post-intervention in all cases except for one sibling who reported a decreased level of impact post-intervention. Pre- and post-intervention self- and parent-report sibling SDQ Total Difficulties Scores indicated reduced difficulties were reported by one sibling and by one parent. In all other cases, increased difficulties were reported post-intervention. One parent wrote a comment on their post-intervention SDQ that the sibling was having investigations into a physical health problem currently “which could contribute to her feelings”.

### PedsQL™4.0

Pre- and post-intervention self-report sibling PedsQL™4.0 Psychosocial Health Summary Scores indicated increased psychosocial health in one case, with five siblings reporting decreased psychosocial health post-intervention. Post-intervention, two parent-report PedsQL™4.0 Psychosocial Health Summary Scores indicated increased psychosocial health. Four parent-reports indicated reduced psychosocial health post-intervention. One parent’s written comment on their answer sheet stated “following [the sibling’s] parents’ evening at school, I am aware of things that I previously wasn’t – had we had parents evening before the first [support group] session my answers on the previous one [pre-intervention PedsQL™4.0] would have been different”.

### Common themes from sibling support group sessions

#### Problems with school

All six siblings said they had difficulty getting to school on time because of their brother/sister’s difficulties and one of them sometimes missed whole mornings of school. Two siblings’ teachers were unaware of their brother/sister’s difficulties, and the siblings were for this reason punished inappropriately for being late to school. Their parents were not aware this was happening.

Four siblings described they lost concentration at school because they have so much on their mind. They described i) worrying about their brother/sister and their parents, ii) staring into space, staring out of the window and missing some of the lesson, iii) not remembering what was said because they weren’t listening, iv) being unable to concentrate in exams, and v) that their grades are affected. One sibling described that because she is disturbed by her brother at night, she is tired at school stating “I’m just too tired to get out of bed because my brother keeps me up nearly every single night…running up and down the stairs and flushing the toilet…once it was 3 a.m. that I actually got to sleep”. They described doing homework at lunchtime in school time because it was very difficult to have space at home to do it free of noise or somewhere they would not be disturbed.

#### Aggression and risk behaviours

Four siblings described the physical strength of their affected brother/sister. For example, one stated their brother was capable of smashing double glazing and that they get hit with that same strength. Another described their brother was physically able to force their father to the ground, and a third sibling said her brother was physically aggressive towards her. Four of the siblings also stated their sibling had threatened them with a knife, or they had witnessed them threaten their parent with a knife. One sibling described that her brother threatened to kill her and had held a knife up to her father. Another sibling recounted that her brother had repeatedly threatened to kill himself using sharp objects and that she found it really frightening. One sibling noted that her parent had to explain to her they must hide sharp objects from her sister because she had threatened to kill herself. Five siblings described their brother/sister was at increased risk of injuries or had injuries such as broken limbs due to them seeming to be ‘fearless’ or not learning from previous mistakes.

#### Danger to pets

Three siblings described their brother or sister had eaten their pet goldfish or pet insects; one sibling stated “my sister ate my goldfish because she could not understand the difference between fish and fish-fingers and my brother can’t understand that children can go down slides but he doesn’t understand that you can’t put animals down the slide”. The child noted they couldn’t have pets anymore due to their brother/sister not understanding how to behave towards pets. Another sibling voiced the fear that their brother would harm their pet gerbil and for that reason they are more vigilant when their brother is nearby.

#### Sense of responsibility

One sibling stated they felt responsible for keeping their brother safe and two others stated they supervise the younger children when their affected brother/sister needs intensive supervision by their parents. However, two siblings stated they were the youngest or the “baby of the family” and didn’t feel responsible when dangerous incidents occurred.

#### Teasing and bullying

The siblings discussed how to manage when others tease their brother/sister. Three siblings in the group described they respond by pointing out their brother or sister has disabilities and that is why they behave differently. This worked for two of the siblings but in one case the teasing continued and they began making fun of the sibling too. One sibling explained she takes a different route home from school to avoid people who tease her about her brother, and one sibling stated she would be embarrassed if the whole class were told about her brother.

#### Witnessing psychotic symptoms

Two siblings had witnessed psychotic symptoms. One sibling described “Once he was on the floor imagining he was getting strangled by an imaginary person”. She said she gets scared when this happens and panics. The other sibling described “he was in the car and, um, he was sitting next to me, and like all of a sudden he just started talking to the chair and I went ‘[brother’s name] who are you talking to?’ And he went ‘um I don’t know but um, I’m trying to beat him up so can you be quiet’”.

#### Lack of shared humour

When asked, two siblings could not think of any occasion when their brother/sister had made them laugh (there being no time-limit on when it could have happened).

#### Hoping for a cure

Two siblings stated they hoped a cure would be found, and the other four siblings wanted their brother/sister to be able to live a more normal life.

### Sibling Evaluation Questionnaire

#### Preferences for audio-conferencing or F2F

Four siblings reported they found audio-conferencing easy/very easy to use and one sibling preferred it over meeting F2F. Two siblings stated it was good not to have to travel. Two reported they found it difficult/very difficult due to the occasional technical hitch such as poor sound quality, a sibling’s line cutting off accidentally, difficulty distinguishing who was talking, too much noise in the house or interruptions from family members at home. One sibling felt she could not get her point across properly and stated “In the phone groups, you can’t really interact with people as much. You can’t say ‘hey, do you want to come and play with me?’, well just, it’s just like a chat, it’s not exactly like a group”. None of the siblings faulted anything about the F2F sessions. Comments included “We got to see each other and distinguish who was talking”, “You could see and hear everyone”, “I loved it”, “wonderful”, “it was good because we get to see everybody”.

#### Benefits of the sibling support group

Sibling-reported benefits from the support group included reduced isolation, building friendships and talking openly, stating “I’ve learnt I’m not alone”, “I’ve made new friends like me”, “it has helped me”, “knowing there are other people [like me]” and “[it’s] a chance to talk about problems freely”.

#### Changes siblings would have made to the sibling support group

All except one sibling said they wanted more sessions overall and fewer audio-conferencing sessions. One sibling stated they would not have changed anything about the sibling support group.

### Parent Evaluation Questionnaire

#### Preferences for audio-conferencing or F2F

Four siblings’ parents found it difficult to enable their child to attend the F2F sessions due to having difficulty being back home in time to bring them to the session, having to ensure their partner or other carer could look after the other child(ren) at home or because they were tired. The advantages of audio-conferencing reported by parents were i) there being no need to travel to hospital, ii) less time and financial cost, iii) less rushing or disruption to their routine, iv) no need to arrange childcare for the other children, v) the siblings did not miss any school, and vi) they were able to get to bed on time as no travel time was needed after the audio-conferencing session. One parent stated “I could focus on the other children whilst she was occupied with the call”. There were two cases of a parent and sibling concurring in their preference that all sessions should be F2F. Despite noting the long distance to travel and the difficulties organising care for other children at home, two parents considered the F2F sessions were preferable for the siblings.

#### Benefits of the sibling support group

Three parents reported an increase in their own awareness of the siblings’ needs and three parents indicated a reduction in the siblings’ feelings of isolation. Five commented that the sibling enjoyed being part of a group of other children who understand their situation and how they feel; with one parent stating “I think [the sibling] felt valued – that their feelings and opinions were/are worth something”. Another parent described the benefit being “that people are bothered about how her brother’s problems affect her life as well [as helping her brother] and trying to help [the sibling] understand how to deal with it”.

#### Changes parents would have made to the sibling support group

Two parents commented that they would have liked the FTF sessions to start at an earlier time of day. One parent thought the activities organised for the support group sessions were good but would have liked more information about them beforehand. One parent regretted that their child had to miss a session due to a pre-arranged school trip. One parent reported they would have liked a parent support group to run at the same time as the sibling support group.

From parents’ and siblings’ evaluations and from researchers’ observations through conducting the study, some advantages and disadvantages of meeting F2F or meeting via audio-conferencing are summarised in Table 3.

**Table 3:** **Advantages and disadvantages of meeting face-to-face and of meeting via audio-conferencing**

**Advantages**	**Disadvantages**
**Face-to-face**	**Face-to-face**
F2F meetings are physically ‘time away’ from their brother or sister for siblings; siblings are the focus of attention (S^1^, P^2^)	Depending on when the group is scheduled to take place, siblings might need to take time out of school, and parents might need to take time off work, particularly if having to travel a long distance (S^1^, P^2^)
Allows full sociability including spontaneous social exchange such as spontaneous social gestures, sharing (such as sharing snacks or passing things to one another when taking part in activities) (F^3^, S^1^)	There is a need for parents to organise care for the affected brother/sister and perhaps for other siblings at home (P^2^)
Facilitators/therapists are therapeutically attending to the ‘whole person’ as opposed to what can be ascertained by voice only (F^3^)	It requires change in the routine in families where there is a need to manage high levels of stress in circumstances where maintaining a routine is important (F^3^)
Siblings had the opportunity to socially ‘lean’ towards or socially link with other members in the group with whom they have more affinity (S^1^)	
**Audio-conferencing**	**Audio-conferencing**
AC can be advantageous as a less physically confrontational option of meeting with other siblings than a F2F meeting (F^3^)	There is reliance on communication being voice-led (F^3^)
Overcomes the barrier of geographical distance (F^3^, P^2^)	There is the potential for technical problems such as accidental ‘cutting off’, or poor quality of sound (S^1^, P^2^, F^3^)
There is no need to travel (F^3^, P^2^)	There is the strict need for one person talking at a time, even in unstructured group ‘chat-time’ (F^3^)
There are no transport costs (F^3^, P^2^)	There are no visual cues as to the meaning of what is not being said or what silences mean (F^3^)
Allows there to be a focus on information-exchange (F^3^)	Participants do not have access to all the means of getting across what they mean to ‘say’ (S^1^, F^3^)
Siblings can access therapeutic assistance in the privacy and comfort of their own home and might therefore feel more at ease and relaxed than they do when they are in an unfamiliar setting (F^3^)	There is the potential for misunderstanding due to the lack of non-verbal communication and the potential to overlook the need to ensure clarification if it occurs (F^3^)
	Participants have no opportunity to communicate with other participants without it being ‘exposed’ to the whole group (S^1^)

^1^S = sibling report, ^2^P = parent report, ^3^F = facilitator observation.

### Individual follow-up interviews

Five of the six siblings and their parents attended a follow-up interview. Any questions that parents and siblings had were addressed. Parents stated that they were not fully aware before the sibling support group of the impact of the affected brother/sister’s behavioural difficulties on the sibling. Three parents considered this was because the busyness of their lives had prevented them from having time to reflect and realise the effect of the child’s behavioural problems on their sibling. A reason one parent gave for not sharing information with the sibling was that they did not want to burden them with it. However, one sibling considered it was worse not being told what was going on as her imagination “runs wild”. One sibling’s parents described a period of three weeks whereby the sibling was quiet and withdrawn commencing towards the end of the support group. This was considered to have perhaps been due to dissociation of having consciously addressed aspects of her brother’s behaviour that she had not done before. She was since behaving as she used to; making new friends, having started a new school year.

It was necessary to refer three of the siblings to their General Practitioner (family doctor) for further support due to the identification of symptoms during the group meetings indicating the possibility of underlying depression, post traumatic stress disorder symptoms or ADHD. Four of the five siblings who attended a follow-up interview said they wanted to stay in touch with at least one sibling from the support group.

## Discussion

This study has involved detailed exploration of the feasibility of audio-conferencing for facilitating sibling support groups to enable children and young people who have a brother or sister with complex neurodevelopmental disorders to effectively access social support. Through this process, when describing their concerns, siblings gave clear descriptions of their brother/sister’s behavioural difficulties in line with those reported by their parents. These findings indicate that siblings as young as eight years old can be reliable informants in the clinical context and where appropriate, siblings could contribute to clinical interviews.

Post-intervention SVQs revealed a reduction in the severity of their concerns resulted from receiving psycho-education, developing coping mechanisms or through feeling empowered to tell their parents about their concern. The sessions which focused on these aspects were audio-conferencing sessions. This can be considered, together with sibling-reported positive outcomes resulting directly from the intervention, as demonstrating the feasibility of audio-conferencing for facilitating sibling support groups.

When considering the people siblings spoke to about their concerns, the number and range increased post-intervention. This included four of the siblings talking to at least one other sibling support group member outside of the support group sessions and is corroborated by answers in the Evaluation Questionnaire whereby siblings reported they had made new friends at the support group. One sibling described how relatives ask how they are, rather than ‘just’ how their brother is. This may be due to relatives being aware of them attending a support group, and that in turn highlighted to the relative(s) the importance of the sibling’s wellbeing.

Siblings’ engagement with the group sessions was extremely high, and feasibility of audio-conferencing was demonstrated through this as well as in further respects. It was not problematic to ensure that every sibling had equal opportunity to speak in both the F2F sessions and the audio-conferencing sessions and there was also little need to prevent siblings talking over one another. As well as reporting concerns relating to their brother or sister’s challenging behaviour within the pre-intervention interview, siblings were able to share some of these experiences in audio-conferencing and F2F sessions.

Additionally, the session focusing on school matters revealed that some parents and teachers were not aware of some of the challenges the siblings faced in their home lives which could negatively affect their experiences at school and their academic performance. This indicates the support group provided a space within which siblings felt able to share difficult experiences, when they had not done so before, such that these could then be addressed. In addition, discussions in the support group sessions enabled siblings’ feelings of isolation to be reduced due to them learning they’re not alone in their situation.

An important aspect of this study was the integration of siblings’ and parents’ evaluations. Whilst the technical feasibility of using audio-conferencing to facilitate sibling support groups was demonstrated, siblings reported a preference for meeting F2F. Sibling reports suggested this was explained by them valuing being physically present to engage in playful interaction and being allowed the choice of having a conversation with other siblings that wouldn’t automatically be exposed to the whole group. It can also be suggested that the absence of non-verbal cues could be more important for children than it is for adults meeting via audio-conferencing, given that children are still developing cognitively, intellectually, socially and emotionally. Despite there being a preference for F2F contacts, the audio-conferencing contacts were adequate, feasible and sufficient, with all six siblings and their parents valuing the support group.

Although the sample size was not suitable for quantitative statistical analyses, some quantitative observations appear appropriate and useful for the development of future research. Increased SDQ Impact Scores, increased Total Difficulties Scores and reduced PedsQL™ 4.0 Psychosocial Health Summary Scores post-intervention could be explained by a number of observations. Three parents stated in their Evaluation Questionnaire that they were not aware beforehand of the impact on the sibling. This suggests that their pre-intervention report would have reflected a more realistic picture i.e. higher impact and a higher level of difficulty. Similarly, additional written comments by parents assist us to identify possible explanations for the increased difficulty reported post-intervention. In one case, for example, a parent noted that a contemporaneous health issue the sibling was dealing with could be influencing their feelings at the time of them completing the post-intervention SDQ. In the case of another sibling, the parent stated on the post-intervention PedsQL™4.0 that they would have scored the pre-intervention version differently if they had been more aware at that stage about the sibling’s situation. This indicates that their post-intervention responses were more realistic than their pre-intervention responses were.

Furthermore, this was the first organised contact (or series of contacts) that the siblings had with health care professionals to consider their own needs and two siblings each reported they had never spoken to anyone about one of their concerns before the support group. By the end of the intervention, the participants recognised the gravity of the situation with regard to their brother or sister, realising that the neurodisability was chronic and that their brother or sister was unlikely to recover completely, participants hence sharing the ‘real picture’ more accurately. Siblings’ feedback following psycho-education however indicated that they felt more prepared because they understood the chronic neurodisability better.

### Limitations

Although this study has contributed novel information about using audio-conferencing to facilitate sibling support groups, further research is required to overcome the limitations and increase the generalizability of results. Generalizability is limited due to the small sample size. Moreover, it is acknowledged that there would be challenges for children who have hearing difficulties or who speak different languages to take part in a similar study. For this reason careful consideration should be given to such aspects in different contexts. Two design elements that are not included in the present study would strengthen the evaluation of the outcomes of the intervention. Participants were a convenience sample and willing participants, which could bias results in a way that a randomised controlled trials design would eradicate. This would also include a control group allowing interpretation of the results as a function of treatment.

## Conclusions

Previous research has underlined the potential value of support groups for siblings of children with chronic illness or disability. This study has demonstrated that audio-conferencing is a feasible and effective means of facilitating sibling support groups to overcome geographical barriers to accessing support. Siblings’ social network widened, and problem-solving skills were cultivated, thus enhancing protective factors for strengthening resilience [7]. Wider applications of the provision of group support via audio-conferencing could include other individuals who are isolated, whether they live in rural or urban areas, for example, those who have been displaced [17].

On the basis of the encouraging findings from this study, for a more definitive understanding as to the place of audio-conferencing in facilitating support for siblings, the following recommendations can be derived for future research. In order to increase generalization of results, a larger study with a greater number of participants is necessary. A study using a support and control groups design (including F2F-session-only groups and audio-conferencing-only groups, for example) would enable further exploration of the preference for F2F or audio-conferencing sessions. Identification of the aspects of group therapy that can be delivered successfully using audio-conferencing could enable them to be optimised for application across many vulnerable groups of children, young people and adults.

Based on experience of running focus groups and therapeutic groups with children, it is advisable to hold groups with a maximum of six children in each group. This study showed that it was feasible for six participants to keep track of who’s speaking on the telephone during audio-conferencing sessions. Facilitators should be competent in running support groups, providing psycho-education at an age-appropriate level and trained in handling sensitive information which might be shared by the sibling support group members. Due care must be taken to ensure privacy and confidentiality are maintained when participants are talking on the telephone from the home setting.

Given parents’ feedback, it is also recommended that parallel parent support groups are run alongside the sibling support groups. Additionally, the number of girls and boys making up the group should be considered, to potentially allow similarities and differences to be compared according to gender. Narrower age ranges in each group would be advisable - particularly perhaps for adolescents, to discuss matters with those of a similar developmental age and allow activities to be tailored more closely to meet the needs of the specific age-groups.

This study has looked specifically at a particular group of siblings who encounter clinically significant risk behaviours in their brothers or sisters and has informed clinicians about the extraordinary experiences of siblings of children in this clinical population. It has highlighted the importance of considering siblings’ needs systematically as part of a package of care when assessing their brothers and sisters. In addition, it is suggested that siblings’ unique insight could enrich the process of diagnosis, the provision of treatment and the ongoing monitoring of progress. The views of siblings can have particular relevance when considering school placements for their brother or sister as they are in a unique position to highlight areas which are of concern in relation to their brother/sister’s interactions with other children. This study indicates that sibling support groups should be established as part of routine practice within a whole-family approach and where necessary, links between CAMHS, school counsellors and young carers groups should be strengthened.

## Abbreviations

ASD: Autism Spectrum Disorder
ADHD: Attention Deficit Hyperactivity Disorder
CAMHS: Child and Adolescent Mental Health Services
CIPP: The Centre for Interventional Paediatric Psychopharmacology
DCSF: Department for Children Schools and Families (United Kingdom)
DH: Department of Health (United Kingdom)
F2F: Face-to-face
PedsQL™4.0: Pediatric Quality of Life Inventory™ Version 4.0
PONS: Profile of Neuropsychiatric Symptoms
SDQ: Strengths and Difficulties Questionnaire
SVQ: Sibling's Views Questionnaire
UK: United Kingdom
